# Performance simulation of polymer-based nanoparticle and void dispersed photonic structures for radiative cooling

**DOI:** 10.1038/s41598-020-80490-z

**Published:** 2021-01-13

**Authors:** Jay Prakash Bijarniya, Jahar Sarkar, Pralay Maiti

**Affiliations:** 1 Department of Mechanical Engineering, Indian Institute of Technology (BHU), Varanasi, UP 221005 India; 2 School of Material Science and Technology, Indian Institute of Technology (BHU), Varanasi, UP 221005 India

**Keywords:** Engineering, Mechanical engineering

## Abstract

Passive radiative cooling is an emerging field and needs further development of material. Hence, the computational approach needs to establish for effective metamaterial design before fabrication. The finite difference time domain (FDTD) method is a promising numerical strategy to study electromagnetic interaction with the material. Here, we simulate using the FDTD method and report the behavior of various nanoparticles (SiO_2_, TiO_2_, Si_3_N_4_) and void dispersed polymers for the solar and thermal infrared spectrums. We propose the algorithm to simulate the surface emissive properties of various material nanostructures in both solar and thermal infrared spectrums, followed by cooling performance estimation. It is indeed found out that staggered and randomly distributed nanoparticle reflects efficiently in the solar radiation spectrum, become highly reflective for thin slab and emits efficiently in the atmospheric window (8–13 µm) over the parallel arrangement with slight variation. Higher slab thickness and concentration yield better reflectivity in the solar spectrum. SiO_2_-nanopores in a polymer, Si_3_N_4_ and TiO_2_ with/without voids in polymer efficiently achieve above 97% reflection in the solar spectrum and exhibits substrate independent radiative cooling properties. SiO_2_ and polymer combination alone is unable to reflect as desired in the solar spectrum and need a highly reflective substrate like silver.

## Introduction

Passive radiative cooling is an emerging and renewable means to generate a cooling effect without the consumption of electricity or any other external energy input^1,2^. It requires above 97% reflection in the solar spectrum (0.25–2.5 µm) and maximum emission in the atmospheric window (8–13 µm), which falls in the domain of the electromagnetic field of study^3^. To achieve a maximum reflection in the solar band and emission in the thermal band for passive radiative cooling, naturally available materials alone are not able to achieve the desired reflection and emission simultaneously, so there is a need for metamaterial development. The metamaterial is an engineered structure with the combination of naturally available materials, hence proper design is essential to achieve desired properties specific to an application. Therefore, the simulation is required by considering electromagnetic interaction with the metamaterial before fabrication for the selection of a proper material structure to get the desired emissivity profile for the cooling application. Many numerical techniques are available to simulate the electromagnetic field, including finite difference time domain (FDTD), finite difference frequency domain (FDFD), rigorous coupled-wave analysis (RCWA), Scattering Matrices and transfer matrix method (TMM). However, the FDTD method is a proven and sable technique to solve for a broad range of frequencies with less computational cost, also computationally efficient method^4^.

Nanoparticles distributed in lossless material like polymer are used to radiate and reflect the selective desired spectrum leads to the development of passive daytime radiative cooling devices. Gentle and Smith^5,6^ choose combined SiO_2_ + SiC nanoparticles of size 50 nm in a 25 µm thick PE sheet backed by Al. The SiO_2_ and SiC have phonon resonance peaks lies in the range of 8–10 µm and 10.5–13 µm, respectively makes it possible to emit in atmospheric window simultaneously avoiding the IR peak of ozone. Bao et al.^7^ simulated the coating of two nanoparticle combinations (SiO_2_ + SiC and TiO_2_ + SiC) on Al/black painted Al using FDTD to get a solar spectral response. Zhai et al.^8^ simulated the radiative properties using TMM and RCWA techniques and experimentally demonstrated microspheres of SiO_2_ embedded polymethyl-pentene based daytime radiative cooler. Huang and Ruan^9^ simulated a polymer-based embedded titanium oxide and carbon black nanoparticles double-layer coating photonic structure for solar spectrum reflection and emission in the atmospheric window, respectively and got optimum results at 0.2 µm size of TiO_2_ nanoparticle for a maximum reflectivity of 0.91 in the solar radiation and emissivity 0.95 in the atmospheric window. Atiganyanun et al.^10^ developed a random structure of SiO_2_ microparticles by spray coating deposition and showed that the randomized silica particles strongly scatter the solar spectrum and facilitate the emission of the mid-IR spectrum. Ao et al.^11^ fabricated diffuse radiative cooler with powder of Na_2_ZnPO_4_ as a reflector on polished Al substrate and achieved net radiative cooling. Ma et al.^12^ developed seven layers of SiO_2_ and Si_3_N_4_ based photonic radiative cooler and achieved 8 °C below ambient temperature. Feng et al.^13^ theoretically investigated the emissivity of SiO_2_ nanoparticles embedded in TPX at 8–13 µm spectrum range and found the particle size of 0.3–0.5 µm has higher emissivity at a slab thickness of 200 µm. Yalcin et al.^14^ simulated the SiO_2_ fiber network in the absence of binder, leaving the air cavity between silica structures for the efficient backscattering of solar spectrum and emission of the mid-infrared spectrum and achieved reflectivity above 97% in the solar spectrum. Wu et al.^15^ numerically compared multilayer dielectric pyramid structures with layered structures using FDTD and showed that the pyramid structures are more efficient in the solar spectrum. The above literature review shows that some numerical analyses were performed to predict the emissivity properties of particle dispersed polymer considering the particle size, multilayer and layer thickness effects. Void dispersion in polymer leads to backscattering and hence maximum reflection in solar spectrum. However, with best of the author’s knowledge, no simulation was performed for void or void and particle dispersed polymer structure to predict surface emissive properties and also effects of particle/void distribution pattern and concentration are still needed consideration, which are important for reflection and emission in passive radiative cooling.

Hence, we report here the simulation of the polymer-based void, particle and void + particle dispersed metamaterials for radiative cooling using the FDTD method to predict emissive profile in both solar and thermal bands. Various lossless nanoparticles (SiO_2_, TiO_2_ and Si_3_N_4_) are considered and effects of distribution pattern (parallel or staggered), slab thickness and nanoparticle concentration are analyzed. The emission spectrum is obtained using the Drude–Lorentz susceptibility model to exploit peaks in the extinction coefficient of the complex refractive index (material property, which is solely responsible for the interaction of radiation with the material). Simulation algorithm to predict emissive characteristics and then radiative cooling performance is presented as well. Finally, the cooling performance of the different structure and void/nanoparticle combinations is compared for a typical summer day in India in terms of minimum surface temperature and maximum cooling power.

## Methodology

Various potential material structures to meet the criteria of passive radiative cooling are selected. The arrangements of the structure are the inline and staggered distribution of nanoparticle and air void combination in a polymer. The inline structure is considered for theoretical comparison and a staggered structure replicates the behavior of the random structure. FDTD simulation is performed to evaluate the spectral responses of synthesized material structures with PML boundary conditions at its top and bottom layers and periodic boundary conditions to its lateral sides, which emphasized the material extent in the horizontal direction. Obtained spectral responses are then used to calculate the cooling performance numerically for a typical hot summer clear sky day in Varanasi, India.

### Discretized Maxwell equation

Maxwell’s equation in the time domain and frequency domain is given in Table 1. These Maxwell’s equations are governing equations to study the behavior of material and electromagnetic interaction. The analytical solution of these equations is limited to some simple geometries with the oversimplification of material properties. The numerical approach is preferred to analyze metamaterial structures with complex refractive indices. Despite the various numerical tool available, FDTD is well developed and suitable for a broad range of frequencies, which is the first need of the radiative cooling application; hence the FDTD method is selected for the numerical solution.

**Table 1:** Maxwell’s equation in the time domain and frequency domain.

Time domain	Frequency domain	
$\nabla \times \vec{E} + \frac{{\partial \vec{B}}}{\partial t} = 0$	$\nabla \times \vec{E} + j\omega \mu \vec{H} = 0$	(1)
$\nabla \times \vec{H} - \frac{{\partial \vec{D}}}{\partial t} = \vec{J}$	$\nabla \times \vec{H} - j\omega \mu \vec{E} = \vec{J}$	(2)
$\nabla \cdot \vec{B} = 0$	$\nabla \cdot \vec{B} = 0$	(3)
$\nabla \cdot \vec{D} = \rho$	$\nabla \cdot \vec{D} = \rho$	(4)
$\vec{D} = \varepsilon \vec{E} = \varepsilon_{0} \vec{E} + \vec{P}$	$\vec{D} = \varepsilon \vec{E} = \varepsilon_{0} \vec{E} + \vec{P}$	(5)
$\vec{B} = \mu \vec{H} = \mu_{0} \vec{H} + \vec{M}$	$\vec{B} = \mu \vec{H} = \mu_{0} \vec{H} + \vec{M}$	(6)

**Figure 1:**
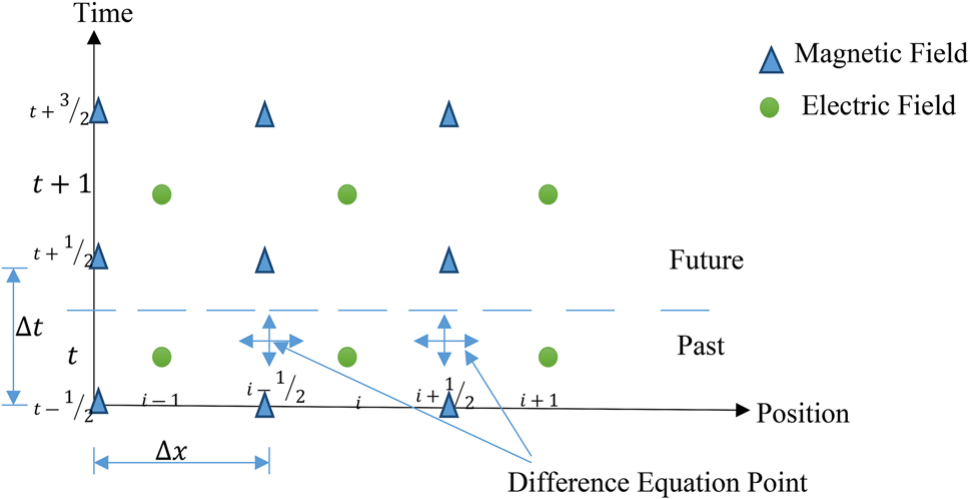
Yee grid arrangement in the x-direction.

The discretized form of time-domain equations in the staggered Yee grid, as in Fig. 1^16^, is given in Eqs. (7–13). The Yee grid is an arrangement for the discretization of Maxwell’s equation in the time domain in order to calculate the electric and magnetic fields at the same time and space points.

7 $$\frac{{E_{z} |_{t}^{i,j + 1,k} - E_{z} |_{t}^{i,j,k} }}{\Delta y} - \frac{{E_{z} |_{t}^{i,j,k + 1} - E_{z} |_{t}^{i,j,k} }}{\Delta z} = - \mu_{xx} |^{i,j,k} \frac{{H_{x} |_{{t + \frac{\Delta t}{2}}}^{i,j,k} - H_{x} |_{{t - \frac{\Delta t}{2}}}^{i,j,k} }}{\Delta t}$$

8 $$\frac{{E_{x} |_{t}^{i,j,k + 1} - E_{x} |_{t}^{i,j,k} }}{\Delta z} - \frac{{E_{z} |_{t}^{i + 1,j,k} - E_{z} |_{t}^{i,j,k} }}{\Delta x} = - \mu_{yy} |^{i,j,k} \frac{{H_{y} |_{{t + \frac{\Delta t}{2}}}^{i,j,k} - H_{y} |_{{t - \frac{\Delta t}{2}}}^{i,j,k} }}{\Delta t}$$

9 $$\frac{{E_{y} |_{t}^{i + 1,j,k} - E_{y} |_{t}^{i,j,k} }}{\Delta x} - \frac{{E_{x} |_{t}^{i,j + 1,k} - E_{x} |_{t}^{i,j,k} }}{\Delta y} = - \mu_{zz} |^{i,j,k} \frac{{H_{z} |_{{t + \frac{\Delta t}{2}}}^{i,j,k} - H_{z} |_{{t - \frac{\Delta t}{2}}}^{i,j,k} }}{\Delta t}$$

10 $$\frac{{H_{z} |_{{t + \frac{\Delta t}{2}}}^{i,j,k} }-{H_{z} |_{{t + \frac{\Delta t}{2}}}^{i,j - 1,k} }}{\Delta y} - \frac{{H_{y} |_{{t + \frac{\Delta t}{2}}}^{i,j,k} - H_{y} |_{{t + \frac{\Delta t}{2}}}^{i,j,k - 1} }}{\Delta z} = \frac{{D_{x} |_{t + \Delta t}^{i,j,k} - D_{x} |_{t}^{i,j,k} }}{\Delta t}$$

11 $$\frac{{H_{x} |_{{t + \frac{\Delta t}{2}}}^{i,j,k} - H_{x} |_{{t + \frac{\Delta t}{2}}}^{i,j,k - 1} }}{\Delta z} - \frac{{H_{z} |_{{t + \frac{\Delta t}{2}}}^{i,j,k} - H_{z} |_{{t + \frac{\Delta t}{2}}}^{i - 1,j,k} }}{\Delta x} = \frac{{D_{y} |_{t + \Delta t}^{i,j,k} - D_{y} |_{t}^{i,j,k} }}{\Delta t}$$

12 $$\frac{{H_{y} |_{{t + \frac{\Delta t}{2}}}^{i,j,k} - H_{y} |_{{t + \frac{\Delta t}{2}}}^{i - 1,j,k} }}{\Delta x} - \frac{{H_{x} |_{{t + \frac{\Delta t}{2}}}^{i,j,k} - H_{x} |_{{t + \frac{\Delta t}{2}}}^{i,j - 1,k} }}{\Delta y} = \frac{{D_{z} |_{t + \Delta t}^{i,j,k} - D_{z} |_{t}^{i,j,k} }}{\Delta t}$$

13 $$D_{x} |_{t}^{i,j,k} = \left( {\varepsilon_{xx} |^{i,j,k} } \right)E_{x} |_{t}^{i,j,k} ,\;D_{y} |_{t}^{i,j,k} = \left( {\varepsilon_{yy} |^{i,j,k} } \right)E_{y} |_{t}^{i,j,k} ,\;D_{z} |_{t}^{i,j,k} = \left( {\varepsilon_{zz} |^{i,j,k} } \right)E_{z} |_{t}^{i,j,k}$$

These discretized equations are further reduced to two-dimension and are considered a constant field in the z-direction. For radiative cooling material, reflectivity and emissivity are important parameters. From the discretized equations, the time-dependent field could be recorded on either side of the material as a transmitted and reflected intensity after the source excitation. Further Fourier transform has been taken to convert the time-dependent fields into the frequency field. This frequency-dependent field is then normalized with the source to calculate absolute reflectivity and transmittance. As transmittance in the solar spectrum is not a desirable property for radiative cooling, it is minimized from the designed structures by introducing more particles in terms of size, concentration and slab thickness. For absorptivity in the thermal band, loss property is included in material and absorbed power is normalized with the source. From Kirchhoff’s law, the emissivity could be estimated as absorptivity as these are the same for a particular wavelength.

### FDTD

The FDTD code is developed for the Python programming interface in MEEP^17^ open-source software is initially used. The larger simulation is performed at the nanoHubmeep tool, which uses scheme as a programming interface and parallel computation with multiple processors, also used wherever required^18^. A Gaussian pulse contains a wide range of frequencies from DC to some maximum frequency, but the DC component has the highest energy in the Gaussian source and generates some artifacts like charge on the grid. An alternative to the Gaussian pulse source is Ricker Wavelet, which is equivalent to the second derivate of Gaussian pulse, which also has a wide range of frequencies and without a DC component. FDTD is a solution of discretized Maxwell’s equations in the time domain and then Fourier transformed to get a spectral response with PML boundary condition and normal incidence of TF/SF source.

### Material dispersion

Material dispersion is modeled using Drude–Lorentz susceptibility to account for loss and emission in the thermal infrared region, as described in Eq. (14)^19^.

14 $$\tilde{\varepsilon }\left( \omega \right) = \varepsilon_{\infty } + \mathop \sum \limits_{p = 1}^{n} \frac{{\Delta \varepsilon_{p} \omega_{p}^{2} }}{{\omega_{p}^{2} - \omega^{2} - 2i\omega \gamma_{p} }}$$

where $\tilde{\varepsilon }\left( \omega \right) = \varepsilon_{r} + j\varepsilon_{i}$ is complex dielectric constant, $\Delta \varepsilon_{p}$ is conductivity and $\gamma_{p}$ is damping factor and complex refractive index is defined as $\tilde{n}\left( \omega \right) = n + jk$.

Material with lossless properties could be directly included in the discretized equation with properties of refractive index as $\varepsilon \mathrm{and} \mu$ parameters, a diagonal tensor in our formulation. The material dispersion with the D–L model is in the frequency domain and needs to be converted into the time domain first to make compatible with the discretized equation and then FDTD simulation is performed.

### Cooling performance estimation

The heat transfer calculations of the developed radiative cooler are described using Eqs. (15–21).

15 $$P_{cool} \left( {T_{s} } \right) = P_{rad} \left( {T_{s} } \right) - P_{atm} \left( {T_{amb} } \right) - P_{solar} - P_{conv + cond} \left( {T_{s} ,T_{amb} } \right)$$

16 $${\text{where}},\quad P_{rad} \left( {T_{s} } \right) = 2\pi \mathop \smallint \limits_{0}^{{\frac{\pi }{2}}} \sin \theta \cos \theta \mathop \smallint \limits_{0}^{\infty } I_{B} \left( {T_{s} ,\lambda } \right)\varepsilon \left( {\lambda ,\theta } \right)d\theta d\lambda$$

17 $$P_{atm} \left( {T_{amb} } \right) = 2\pi \mathop \smallint \limits_{0}^{{\frac{\pi }{2}}} \sin \theta \cos \theta \mathop \smallint \limits_{0}^{\infty } I_{B} \left( {T_{amb} ,\lambda } \right)\varepsilon \left( {\lambda ,\theta } \right)\varepsilon_{atm} \left( {\lambda ,\theta } \right)d\theta d\lambda$$

18 $$I_{B} \left( {T,\lambda } \right) = \frac{{2hc^{2} }}{{\lambda^{5} \left( {e^{{\frac{hc}{{\lambda kT}}}} - 1} \right)}}$$

19 $$\varepsilon_{atm} \left( {\lambda ,\theta } \right) = 1 - t\left( \lambda \right)^{{\frac{1}{\cos \theta }}}$$

20 $$P_{solar} = \mathop \smallint \limits_{0}^{\infty } I_{solar} \left( \lambda \right)\varepsilon \left( {\lambda ,\theta_{solar} } \right)d\lambda$$

21 $$P_{conv + cond} \left( {T_{s} ,T_{amb} } \right) = h_{c} \left( {T_{amb} - T_{s} } \right)$$

Here $P_{cool}$ is net cooling power at radiative cooler surface temperature ($T_{s}$) and ambient temperature ($T_{amb}$). Details are given in an earlier publication^20^.

The calculated solar spectrum then further processes to obtain cooling performance in terms of maximum temperature drop at cooling power equals to zero, and maximum cooling power at radiative cooler temperature equals to ambient temperature.

### Simulation algorithm

**Figure 2:**
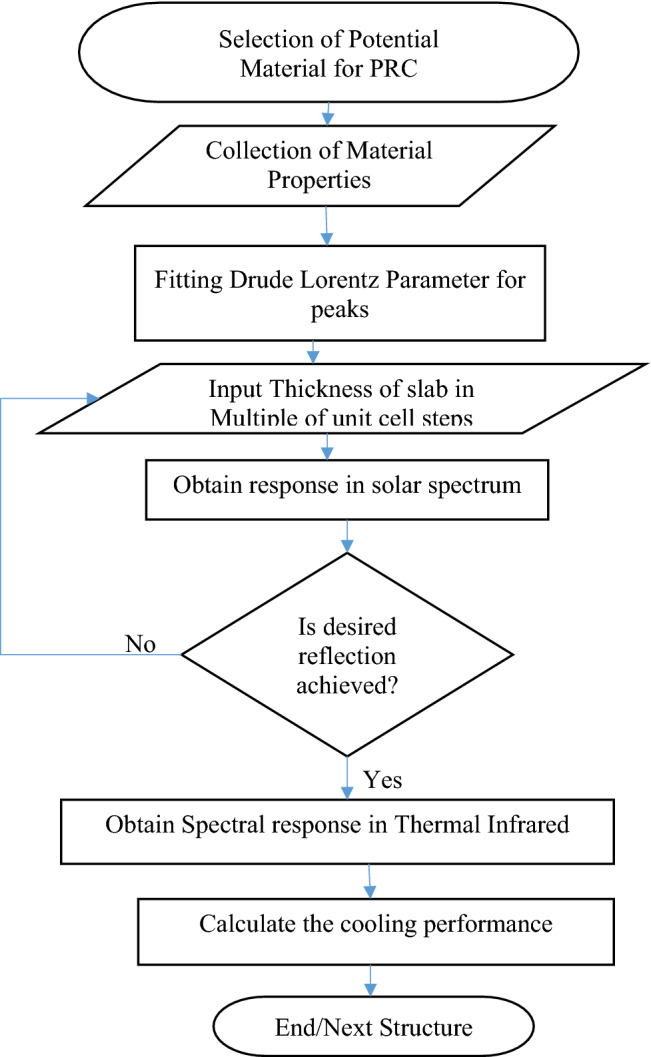
Simulation algorithm.

The flow chart is shown in Fig. 2. FDTD method involves a rectangular grid and to minimize dispersion, gird resolution must be at least less than ten times of wavelength for convergence grid resolution refined until two successive similar results.

For both inline and staggered structures, the solar spectrum response is observed and the thickness of the slab increases till the desired reflection is achieved. Then the further spectral response is observed in thermal infrared for the thickness of the slab obtained in the solar spectrum. These spectral responses are used to calculate the radiative cooling parameter for the selected location Varanasi, India, summer weather.

## Material for passive radiative cooling

Here three types of polymer-based metamaterial structures are considered; (1) nanoparticle dispersed, (2) air void dispersed and (3) nanoparticle-void dispersed. The refractive index of nanoparticles or base material is important for passive radiative cooling. Resonance and extinction of material have been used for modeling the emissive nature in the thermal infrared spectrum^21^. Solid materials are structurally categorized into layered and randomly distributed structures. Layered material structures have costly manufacturing as it involves manufacturing methods like E-Beam evaporation, sputtering, etc. The other structures of material are the distribution of nano/microparticles into the polymer, stand-alone nanoparticles with some binder^22,23^. PVDF polymer has been taken as the base material as it is lossless in the solar spectrum to achieve reflection that efficiently works for passive radiative cooling. SiO_2_, TiO_2_ and Si_3_N_4_ are considered nanoparticles, which are lossless in the solar spectrum due to high electric permittivity^6,21^. Considered particles are simpler and easier to distribute in the polymer. In this study, the complex refractive index is considered to observe the spectral response in the thermal infrared region. In the solar spectrum, due to the lossless behavior of considered nanoparticles as well as polymer, the real part of the refractive index is sufficient to observe the actual behavior. The abrupt change in the refractive index leads to a mismatch in impedance and causes reflection.

**Figure 3:**
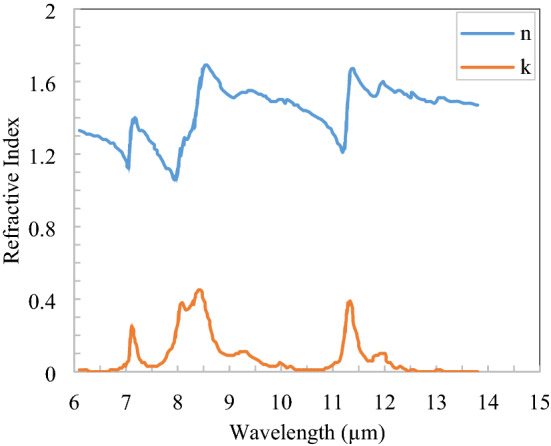
Refractive index of polymer (PVDF) in the thermal infrared spectrum^21^.

The complex refractive index of polymer PVDF is shown in Fig. 3 in the thermal infrared region^21^ and got the three Drude-Lorentz Susceptibility parameter $\varepsilon_{\infty } = 1$, $\Delta \varepsilon_{1} = 0.507$, $\Delta \varepsilon_{2} = 0.091$,$\Delta \varepsilon_{3} = 0$, $\omega_{1} = 0.17$, $\omega_{2} = 0.11$, $\omega_{3} = 0.08$, $\gamma_{1} = 0$, $\gamma_{2} = 0.008$, $\gamma_{3} = 0$ and these parameters are approximately fitted with the refractive index of PVDF given in Fig. 3. For the spectral range of 0.25–2.5 µm, only the real part of the refractive index, i.e., 1.4 is taken into consideration as the imaginary part is negligible^24^.

**Figure 4:**
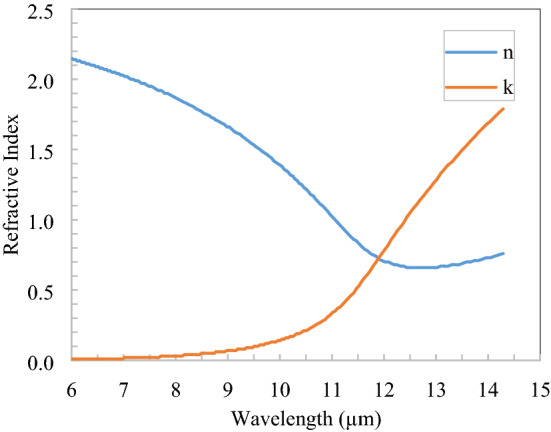
Refractive index of TiO_2_ in the thermal infrared spectrum^25^.

The complex refractive index of TiO_2_ is shown in Fig. 4 in the thermal infrared region^25^ and got the three Drude–Lorentz Susceptibility parameters: $\varepsilon_{\infty } = 1$, $\Delta \varepsilon_{1} = 0.00155$, $\Delta \varepsilon_{2} = 2.5284$, $\Delta \varepsilon_{3} = 0.9632$, $\omega_{1} = 0.096$, $\omega_{2} = 0.0621$, $\omega_{3} = 0.13$, $\gamma_{1} = 0.000081$, $\gamma_{2} = 0.1633$, $\gamma_{3} = 0$ and these parameters are approximately fitted with the refractive index of TiO_2_ given in Fig. 4. In the 0.25–2.5 µm spectral region refractive index of TiO_2_ is 2.3, which has the real part only. TiO_2_ is lossless in 0.4–2.5 µm and hence lossless properties of TiO_2_ are assumed in only a narrow band (0.25–0.4 µm) because the solar flux in this band is low compared to the rest of the spectrum range. Also, 0.25–0.4 µm spectral flux is diluted in the atmosphere due to Rayleigh scattering.

**Figure 5:**
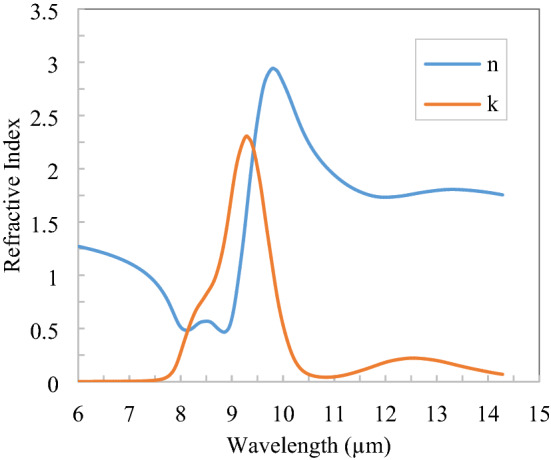
Refractive index of SiO_2_ in the thermal infrared spectrum^25,26^.

The complex refractive index of SiO_2_ is shown in Fig. 5 in the thermal infrared region^26^ and got the three Drude–Lorentz susceptibility parameters: $\varepsilon_{\infty } = 1$, $\Delta \varepsilon_{1} = 0.0816$, $\Delta \varepsilon_{2} = 0.678$,$\Delta \varepsilon_{3} = 0.47$, $\omega_{1} = 0.0805$, $\omega_{2} = 0.1066$, $\omega_{3} = 0.25$, $\gamma_{1} = 0.0058$, $\gamma_{2} = 0.0068$, $\gamma_{3} = 0.1$ and these parameters are approximately fitted with the refractive index of SiO_2_ as given in Fig. 5. As the imaginary part of the refractive index is negligible in the spectral range of 0.25–2.5 µm, only real portion of the refractive index, i.e., 1.53 is considered for SiO_2_^27^. SiO_2_ is lossless in the entire solar spectrum.

**Figure 6:**
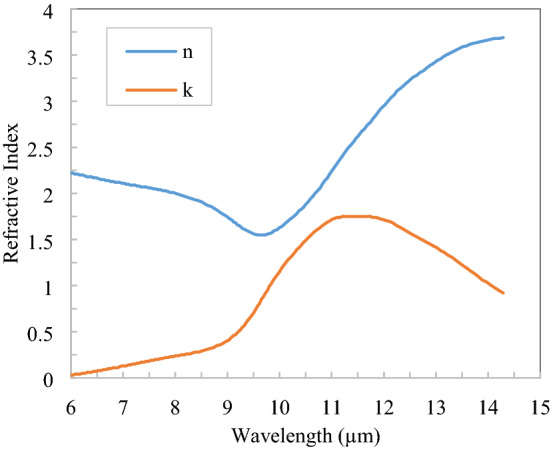
Refractive index of Si_3_N_4_ in the thermal infrared spectrum^25^.

In the spectral range of 0.25–2.5 µm, a real refractive index value of 2.05 is considered for Si_3_N_4_ because the imaginary part is negligible^28^. The complex refractive index of Si_3_N_4_ is shown in Fig. 6 in the thermal infrared region^25^ and got the three Drude–Lorentz Susceptibility parameter $\varepsilon_{\infty } = 1$, $\Delta \varepsilon_{1} = 1.361$, $\Delta \varepsilon_{2} = 3.99$, $\Delta \varepsilon_{3} = 1.88$, $\omega_{1} = 0.0886$, $\omega_{2} = 0.2$, $\omega_{3} = 0.077$,$\gamma_{1} = 0.02$, $\gamma_{2} = 0$, $\gamma_{3} = 0.02$ and these parameter approximately fitted with refractive index of Si_3_N_4_ given in Fig. 6. Si_3_N_4_ also has low imaginary refractive index similar to SiO_2_ with slight higher value. The average value of real refractive index in solar spectrum is assumed due to slight variation and this variation has negligible impact on reflection spectrum.

## Results and discussion

The simulation using the FDTD method at MEEP open-source software^17^ is performed in the solar spectrum (0.25–2.5 µm) and thermal infrared spectrum (6–15 µm) and the spectral response observed for proposed material structures.

**Figure 7:**
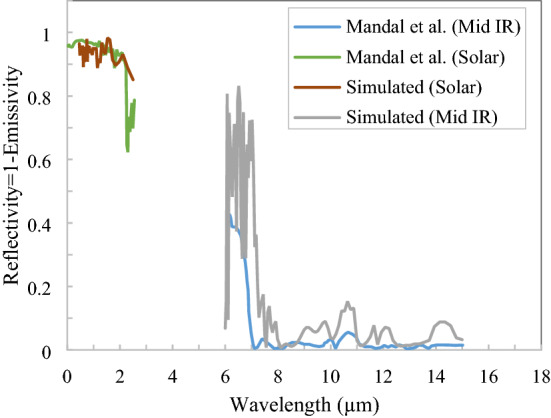
Validation of simulated results with Mandal et al.^21^ for spectral responses.

The validation with the experimental results of Mandal et al.^21^ is performed for spectral responses. The structure of material proposed by Mandal et al.^21^ is a random air void created in a polymer with varying sizes of voids from 50 nm to some micron. The structure validation is created with a void size range of 50 nm–5 µm in the polymer. The refractive index in the solar spectrum is considered only a real portion because the imaginary part is negligible. The real part of the refractive index for air is unity, whereas it is approximately 1.4 for the polymer. In the thermal infrared region, the complex refractive index is taken into account and fitted with the Drude–Lorentz parameter to calculate absorption. The spectral responses of simulated results fairly match with literature reference considered shown in Fig. 7.

We have performed the simulation of the inline and staggered structures of nanoparticles in a polymer that started with a thickness of 25 µm. It increased until the desired reflection in the solar spectrum is achieved. Then the further thermal infrared spectral response is recorded for that thickness.

**Figure 8:**
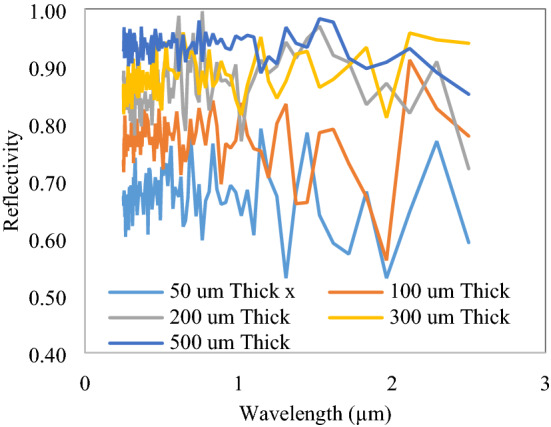
Variation of reflectivity with slab thickness.

**Figure 9:**
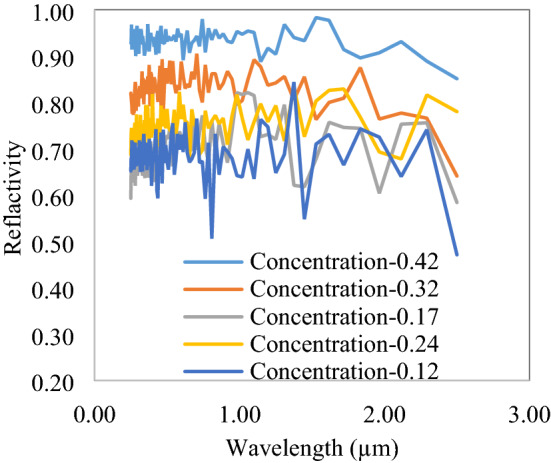
Concentration effect on solar spectrum reflectivity of 500 µm thick slab.

The effect of thickness of the slab is recorded, as shown in Fig. 8. The solar spectrum reflectivity increases from 0.65 to 0.96, with an increase in slab thickness from 50 to 500 µm. A similar variation with the concentration of voids or nanoparticles could also be observed, as shown in Fig. 9. Lower concentration and thickness have a number of peaks and valley due to less interactions of the solar spectrum with voids and particles. The reflectivity curve has straightened at a higher thickness and at 500 µm, it reflects all the solar spectrum with nearly constant reflectivity. It is also noted that a wide range size of lossless particles or voids is important to reflect all solar spectrum constantly. The reflectivity curve has a decreasing trend after 2 µm wavelength due to the considered size of voids and particles, limited in 0.2–6 µm range. The bigger size particles efficiently backscatter the NIR wavelength; however, here, due to constraints of simulation size and time required, particle size is limited to 6 µm only.

The concentration of embedded particles/voids is obtained from the area occupied by the nanoparticles to the total area of the polymer. Here, also, due to more available space between particles at a lower concentration, the solar spectrum penetrates to higher depths and decreases the reflectivity. After the concentration of 0.42, reflectivity reaches the desired value and also structure tightly fits with particles and voids.

### Inline structure

**Figure 10:**
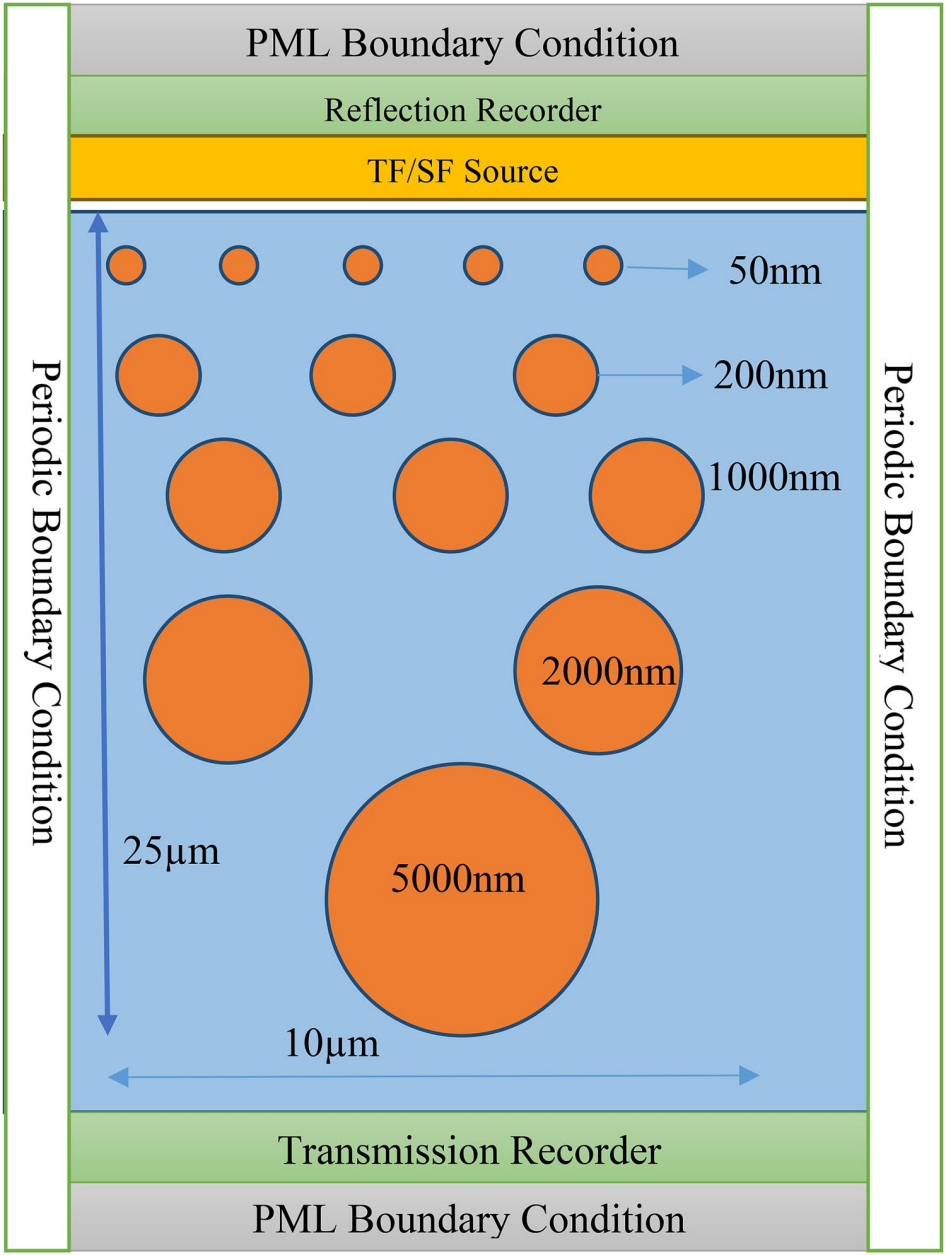
Primitive cell of inline nanoparticle distribution in polymer.

The inline structure design is shown in Fig. 10 with boundary conditions and source used in FDTD. There is five sizes or more type of nanoparticle that is selected in 25 µm thickness. This is the building block of our structure considered as the unit cell or primitive cell. Then the further thickness of the slab increased by adding steps of one block, i.e., 25 µm, till the above 96% reflectivity achieved in the solar spectrum.

**Figure 11:**
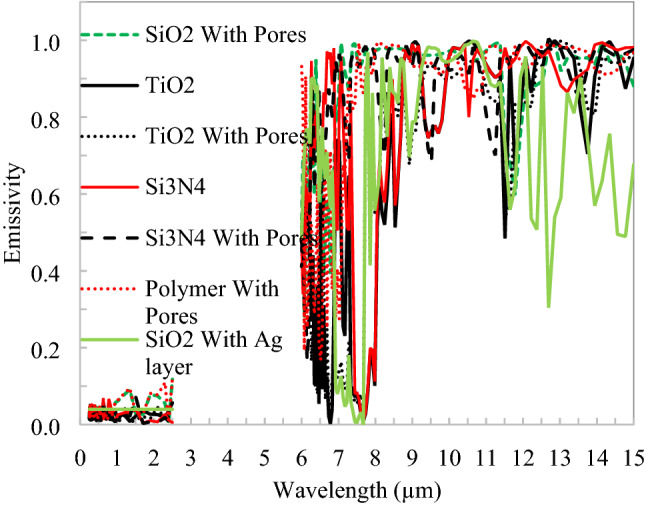
Spectral response of inline distributed nanoparticles and void in a polymer.

The spectral responses of the inline structure shown in Fig. 11 for all seven combinations of nanoparticles (TiO_2_, SiO_2_, Si_3_N_4_) with and without air voids in the polymer. The simulated results need solar spectrum reflectivity is greater than 0.97 and emission in atmospheric window extends to further long wavelengths. At 550 µm thickness of the parallel unit cell of 25 µm, it is achieved above 96% reflection in solar, so here we have 22 building blocks of basic suggested material structure. The SiO_2_ and polymer structure alone insufficient to achieve higher reflectivity in the solar spectrum, so here silver backing is required; however, the other structures are substrate independent.

**Figure 12:**
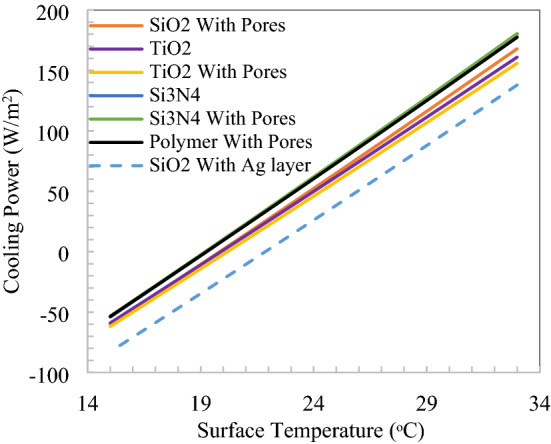
Cooling power v/s temperature of inline structure radiative cooler.

The cooling performance in terms of the surface temperature of radiative cooler and cooling power is shown in Fig. 12. Si_3_N_4_ with void (pores) have better performance than other proposed structures, followed by polymer with pores. SiO_2_ with pores in polymer have broadband emission also relative cooling power. The performance is similar for all nanoparticle/void combinations due to the same location and combined effect of governing parameters, which are emissivity in the solar and thermal infrared spectrums, the emissivity of atmosphere, convection loss. There is a constant difference in cooling power that occurs corresponding to surface temperature due to emissivity in the thermal infrared region only because rest parameters are constant at a particular location for a radiative cooler. The average cooling power difference between SiO_2_ with the Ag layer and Si_3_N_4_ with pores of 35 W/m^2^ signifies the effect of emissivity after achieving the same solar emissivity.

### Staggered structure

**Figure 13:**
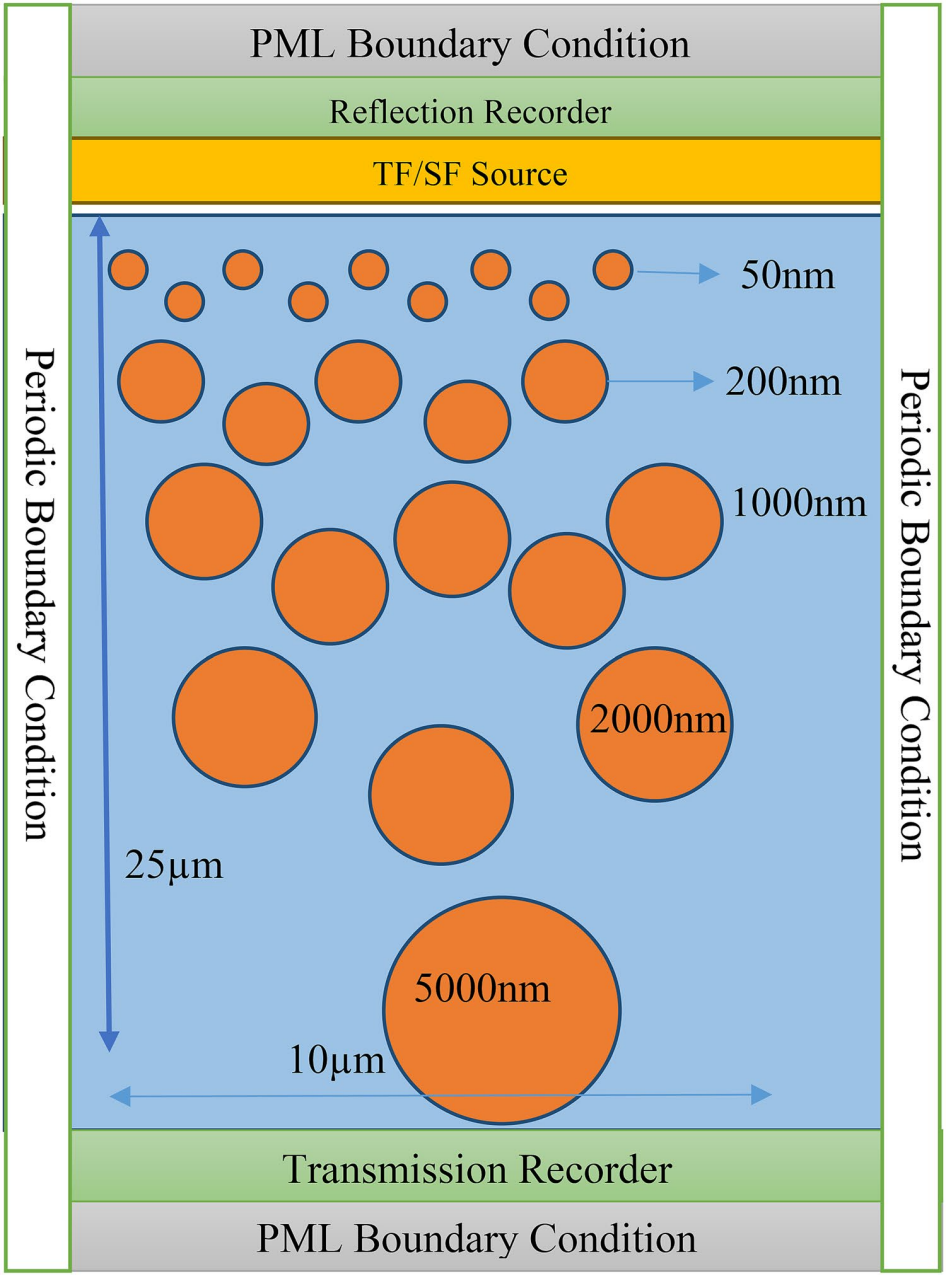
Primitive cell of staggered nanoparticle distribution in polymer.

**Figure 14:**
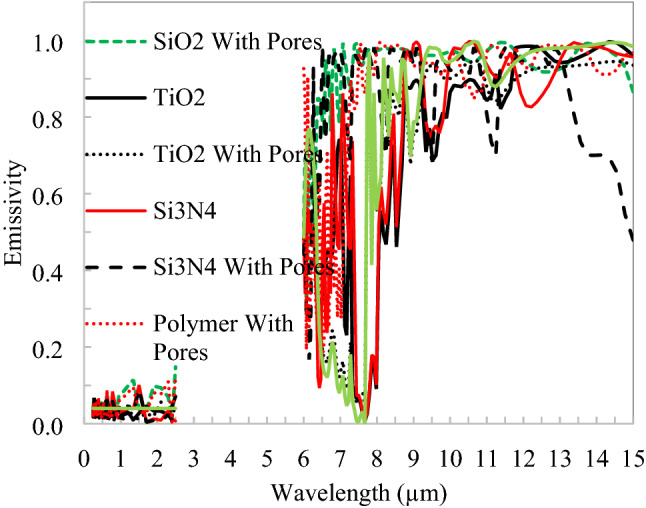
Spectral response of staggered distributed nanoparticles and void in polymer.

The staggered arrangement design in Fig. 13 with required boundary conditions and source represents the unit cell of thickness 25 µm contains five or more sizes of nanoparticles again. The emission spectrum is shown in Fig. 14, which shows that above 96% reflection in the solar spectrum is achieved for thickness greater than 500 µm.

**Figure 15:**
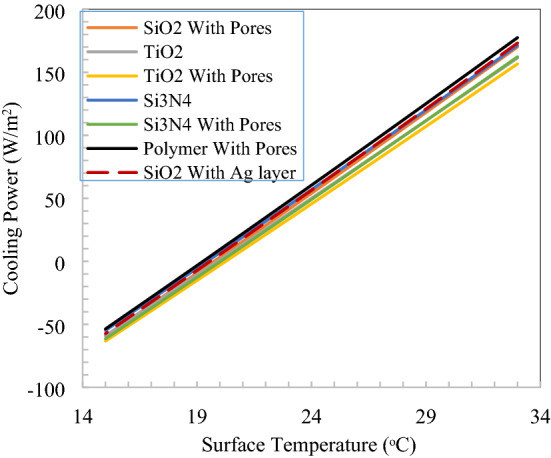
Cooling power v/s temperature for staggered structure.

Spectral responses of nanoparticles and void dispersed in polymer with the staggered arrangement shown in Fig. 14, in the solar spectrum reflectivity, is greater than 0.97 and emission in atmospheric window extends to further long wavelengths. Again at 500 µm thickness of staggered unit cell of 25 µm, 97% solar reflection is achieved, so here we have 20 building blocks of basic suggested material structure. The cooling performance in terms of the surface temperature of radiative cooler and cooling power is shown in Fig. 15. SiO_2_ with the Ag layer has better performance than other proposed structures, followed by polymer with pores. The spectral response of silicon dioxide without a void in polymer for both structures, the calculated reflectivity is too low, it is clear that to achieve radiative cooling and it will further depend on substrate. However, emissivity is quite satisfactory to achieve net emission in the thermal infrared region. Considering both the structures, emissivity has nearly the same pattern in the thermal band and one possible reason could be the structures are thick enough as the emissivity of SiO_2_ nanoparticles saturated at 200 µm thickness. But the solar reflection requirement increases the thickness of the slab^14^. It is observed that silicon dioxide nanoparticle dispersed in polymer alone not sufficient to reflect as desired in the solar spectrum and alternate structure with particle and void may lead to optimum results. SiO_2_ nanoparticle on the surface of polymer with numerous void inside reflects 97% solar spectrum and creates a flexible material for broad ranges of applications^29^. The difference in cooling power raises 6–17 W/m^2^ for a 15 °C rise in the temperature of two extreme performance nanoparticles.

### Comparison

**Figure 16:**
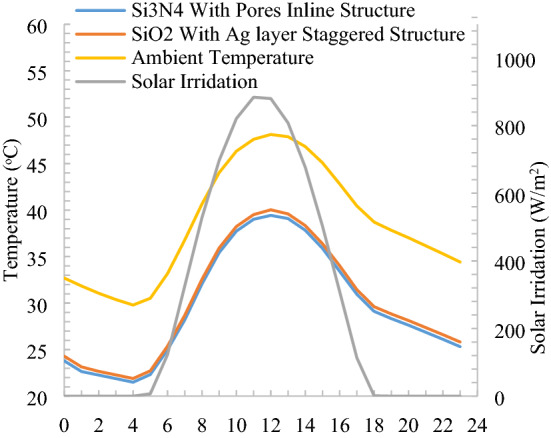
Comparison of surface temperature for selected nanoparticles and structures.

The cooling performance in terms of maximum temperature drop shown in Fig. 16 is calculated for the best-performing structures of inline and staggered each from cooling power v/s surface temperature calculation. A typical hot summer day (1st June) at Varanasi, India, is selected for the minimum temperature drop performance evaluation. From maximum temperature drop of radiative cooler’s performance exhibits that radiative cooler remains sub-ambient through the day and night cycle of 24 h. Both inlined and staggered structures are difficult to fabricate, some nanofabrication processes exist, but it’s hardly useful to fabricate inline structure for passive radiative cooling. Although the differences in solar spectrum reflectivity are negligible, the staggered structure is giving better performance. Moreover, the staggered structure is comparable to random structures and simulated to confirm the randomness also. Hence the random structure (real structure and easy to fabricate) is preferable for a passive radiative cooling application.

Both inline and staggered structures have similar sub-ambient performance due to the same reflectivity in the solar spectrum and a slight difference in thermal infrared emission. The minimum difference occurs in the morning to noontime due to solar and convective heat gain by the radiative cooler. After that, the gap between ambient temperature and radiative cooler temperature widens. The maximum temperature drop from an ambient of 9.5 °C in the evening and early morning observed for inline Si_3_N_4_ with air voids in the polymer at the same time solar flux is nearly zero and wind velocity also reaches moderate. The minimum temperature drop of 7.5 °C is observed for the staggered structure of SiO_2_ with an Ag layer due to solar heat gain and loss of heat in convection due to the wind is observed. Here, we have only a 2 °C difference in temperature drop from ambient due to strange alternative solar and convection heat gain for the selected day. However, minimum temperature drop performance is highly weather parameter-dependent, which may lead to a wider gap between structures with a time of the day.

**Figure 17:**
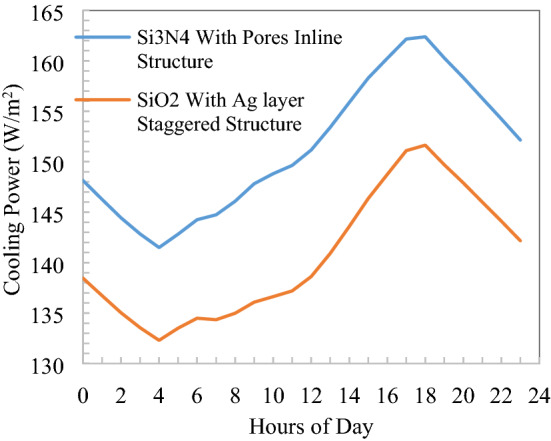
Comparison of cooling power for selected nanoparticles and structures.

Cooling power is calculated at ambient temperature for both parallel and staggered structures as shown in Fig. 17 for a 24 h day and night cycle. There is around 10 W/m^2^ difference of parallel and staggered structures in cooling power, which is 6.5% due to the material construction and emissivity difference in the thermal infrared wavelength. Minimum of 132 W/m^2^ for SiO_2_ with Ag layer staggered structure and maximum of 162 W/m^2^ cooling power is observed for inline structure. Minimum cooling power during the morning to noon time when solar has its peak intensity thereafter, wind velocity and solar intensity reduce leads to an increase in cooling power, which is noticeable. Also, maximum cooling power calculated at radiative cooler surface temperature equivalent to ambient temperature, at the evening time ambient temperature on the higher side which increases the net cooling power and create different pattern than minimum temperature drop from ambient.

## Conclusions

Numerical tools are efficient and cost-effective ways to analyze and design before fabrication. As passive radiative cooling is a new and emerging field, few commercial software is available to simulate. However, techniques like FDTD can be implemented through the developed code or opens source modules. TiO_2_, Si_3_N_4_ dispersed structures tend to reflect above 96% in the solar spectrum at 550 µm thickness due to the wide gap of real refractive index between polymer and particles. In the thermal infrared region, TiO_2_, SiO_2_ and Si_3_N_4_ have peaks of extinction coefficients and efficiently emits selectively through the atmospheric window. For SiO_2_-polymer combination, the desired reflection in the solar spectrum is highly dependent on substrate material even up to 1500 µm thickness as there is transparency, also the narrow difference between real refractive indices. However, SiO_2_-void-polymer combination becomes substrate independent due to differences in refractive indices and atmospheric window emission is as expected due to extinction coefficient peaks near 10 µm wavelength. The difference in the spectrum has minor changes by the introduction of the void in the structure. However, the void creation and size control in the polymer is a difficult task, so it is recommended that nanoparticle and polymer structure is easy to manufacture and efficient for radiative cooling.

The maximum cooling power of 162 W/m^2^ at ambient temperature is observed in a typical hot summer clear day of Varanasi, India, during evening time when solar gain reduces to zero for Si_3_N_4_ with voids inline structure and during peak solar radiation 132 W/m^2^ cooling power is observed for SiO_2_ with the silver layer in staggered structure. As it is a higher side to previously presented results of various locations due to hot summer days, have elevated ambient temperature leads to the emission of fourth power to absolute temperature. A maximum temperature drop of 9.5 °C during night and 7.5 °C during the daytime performance is observed.

We found that two mixed lossless material having a wide difference of real refractive index in the solar spectrum and extinction peaks in thermal infrared are simple to fabricate and potential surfaces for radiative cooling. However, the narrow difference in real refractive index leads to transparent structures and highly dependent on substrate, i.e., SiO_2_ and polymer combination. The reflectivity in the solar spectrum can be increased by increasing the slab thickness or/and the nanoparticle/void concentration.

## Abbreviations

B: Magnetic displacement field
c: Speed of light
D: Electric displacement field
DC: Direct current
E: Electric field
FDTD: Finite difference time domain
h: Plank’s constant
H: Magnetic field
I: Irradiation
J: Current density
k: Stephan Boltzmann’s constant, imaginary part of refractive index
M: Magnetic polarization
MEEP: MIT electromagnetic equation propagation
n: Real part of complex refractive index
NIR: Near infrared
P: Electric polarization, power
PET: Polyethylene terephthalate
PMMA: Polymethyl methacrylate
PVDF: Polyvinylidene fluoride
PML: Perfectly matched layers
T: Temperature
TF/SF: Total field/scattered field
ε: Electric permittivity, complex dielectric constant, emissivity
µ: Magnetic permeability
λ: Wavelength
